# Plasma glutamine concentration after intensive care unit discharge: an observational study

**DOI:** 10.1186/s13054-014-0677-8

**Published:** 2014-12-09

**Authors:** Marie Smedberg, Johanna Nordmark Grass, Linn Pettersson, Åke Norberg, Olav Rooyackers, Jan Wernerman

**Affiliations:** Department of Anesthesiology and Intensive Care Medicine, K32, Karolinska University Hospital Huddinge, 141 86 Stockholm, Sweden

## Abstract

**Introduction:**

Low plasma glutamine concentration at ICU admission is associated with unfavorable outcomes. The prediction of plasma glutamine concentration after ICU discharge on outcomes has not been characterized. In the recent Scandinavian Glutamine Trial, a survival advantage was seen with glutamine supplementation as long as patients stayed in the ICU. It was therefore hypothesized that the glutamine level may drop at ICU discharge, indicative of a sustained glutamine deficiency, which may be related to outcome.

**Methods:**

Fully fed ICU patients intravenously supplemented with glutamine for >3 days were studied at ICU discharge and post ICU. In study A, plasma glutamine level was followed every 5 to 7 days post ICU of the remaining hospital stay and compared to the level on the day of ICU discharge (n = 63). In study B, plasma glutamine level 24 to 72 hours after ICU discharge was related to 12-month all-cause mortality (n = 100).

**Results:**

Post-ICU plasma glutamine levels were within normal range and were not found to be predictive for mortality outcome. Plasma glutamine level at discharge, on the other hand, was within normal limits but higher in nonsurvivors. In addition, it was adding prediction value to discharge SOFA scores for post-ICU mortality.

**Conclusions:**

Post-ICU glutamine levels are not indicative of glutamine depletion. The relation between plasma glutamine concentration and glutamine availability during critical illness is not well understood, and needs to be studied further to define the possible role for glutamine supplementation.

## Introduction

A low plasma concentration of glutamine at intensive care unit (ICU) admission is associated with an unfavorable outcome [1,2]. A high glutamine concentration at ICU admission may also communicate a negative prediction [2]. Other reports claim that free glutamine depletion in tissues (during ICU stays) is associated with unfavorable outcome [3,4]. So the hypothesis - that at least some critically ill patients may have a shortage of free glutamine - seems to be valid. Conventional products for enteral feeding of critically ill patients contain the same amounts of glutamine as ordinary food, while conventional products for parenteral feeding of these patients do not contain any glutamine, related to stability issues. Consequently, supplementation of glutamine was suggested [5].

Literature on glutamine supplementation for critically ill patients is extensive. Meta-analyses suggest a beneficial effect when intravenous (iv) supplementation is given to ICU patients on parenteral nutrition [6,7]. Reports on enteral supplementation to ICU patients on enteral nutrition are less conclusive [6]. Only a few studies include plasma glutamine concentration measurements as a possible indicator of individuals with glutamine depletion, which should be the proper target group for supplementation [8-10]. The rational for supplementing critically ill patients - without knowledge of their actual glutamine status - has been that glutamine supplementation was considered to be without side effects [5,11]. But recently, a study demonstrated harm when supplying glutamine in high doses as a part of a pharmaconutrition concept in the acute phase of critical illness [9], although the subjects were a selected subgroup that received hypocaloric nutrition and had two or more organ failures. So better knowledge of the glutamine status and whether or not an actual deficiency of glutamine is present in the individual patient is needed.

The Scandinavian Glutamine Trial indicated in secondary outcomes of only patients treated per protocol that iv glutamine supplementation may improve outcome during ICU stay [12]. This possible benefit, however, was not sustained after ICU discharge, which may be related to that glutamine supplementation was discontinued at discharge. To elucidate this hypothesis two observational research studies were launched: Study A - to follow plasma glutamine concentrations after ICU discharge, and Study B - to find out if post-ICU plasma glutamine concentration is a mortality predictor.

## Methods

### Patients

**Figure 1:**
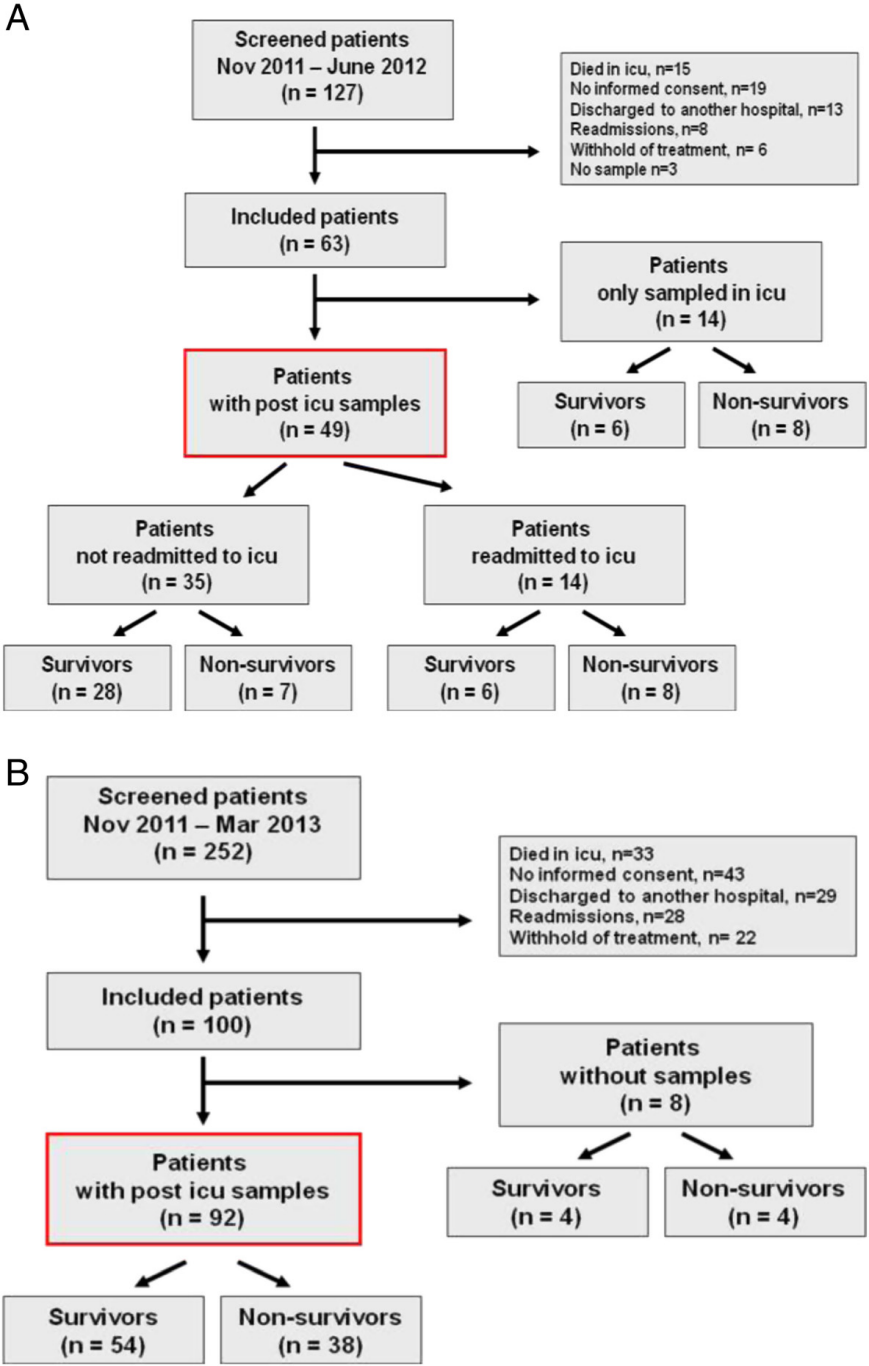
**CONSORT diagrams on patients screened and included in the studies.** Patients admitted to the ICU) and given exogenous intravenous glutamine supplementation together with full nutrition for >3 days were eligible. **(A)** describes patients sampled on their last day of ICU stay and thereafter every 5 to 7 days during the remaining hospital stay. Mortality, dropouts and ICU readmissions are indicated. **(B)** describes patients discharged from the ICU and sampled 24 to 72 hours after discharge. Mortality and dropouts are indicated. ICU, intensive care unit.

The unit has 12 beds and is the only ICU at the Huddinge wing of Karolinska University Hospital, which includes transplant surgery, major gastrointestinal surgery, hematology and infectious diseases, but not cardiac surgery, trauma care and neurosurgery. The protocol was to include consecutive patients admitted to the general ICU at Karolinska University Hospital Huddinge, who received iv glutamine supplement for >3 days. Exclusion criteria were: age <18, restrains of treatment, absence of informed consent, and readmissions to the ICU. Patients already in the study continued their enrollment according to protocol - if they were readmitted. Figure 1 contains CONSORT diagrams on patient recruitment. Before obtaining patients’ written informed consent, patients (or next of kin) were informed verbally and in writing about the study and possible risks. The Regional Ethics Committee in Stockholm approved the protocol, which complied with the Helsinki Declaration.

### Protocols

In study A (post-ICU temporal pattern of plasma glutamine concentrations) an initial sample was taken during ongoing glutamine supplementation in the ICU <24 hours before discharge. Thereafter, samples were taken every 5 to 7 days as long as patients stayed in the hospital. If patients were readmitted to the ICU (and then given iv glutamine supplementation), sampling continued as per protocol.

In study B (post-ICU concentration of plasma glutamine as outcome predictor) a sample was taken between 24 and 72 hours after ICU discharge. All-cause mortality was then recorded for 12 months. Some patients (n = 56) were included in both study protocols.

### Nutrition

During ICU admission the nutritional protocol was aligned with the Scandinavian Glutamine Trial protocol [12]. All patients were started on enteral nutrition as soon as no contraindication was evident. In parallel with the start of enteral nutrition iv glutamine supplementation was started. Complementary parenteral nutrition was started if caloric target (as per indirect calorimetry energy expenditure) was not reached on day 5 of ICU stay. Glutamine supplementation was administered as L-alanyl-L-glutamine 200 mg/ml in 100 mL containers (Dipeptiven, Fresenius, Bad Homburg, Germany), 100 mL per 24 hours if <60 kg, 200 mL per 24 hours if >60 kg, and 300 mL if >80 kg and in addition on continuous renal replacement therapy - as a continuous infusion over 24 hours. Deviations from the Scandinavian Glutamine Trial protocol were (i) postponing of complementary parenteral nutrition until day 5, (ii) constant infusion of the glutamine-containing dipeptide during 24 hours instead of 12 hours, and (iii) dosing of the dipeptide in body-weight intervals rather than per kg.

### Analysis

As described earlier, high-pressure liquid chromatography (HPLC) using an on-column derivatization with ortho-phtaldialdehyde/3-mercaptopropionic acid (OPA/3-MPA) was used to analyze the plasma glutamine concentration [13].

### Statistics

Fifty patients were planned for inclusion in study A (post-ICU temporal pattern of plasma glutamine concentrations) and 100 patients in study B (post-ICU concentration of plasma glutamine as outcome predictor). In study B, data from an earlier study over admission plasma glutamine concentration as an outcome predictor, suggested that n = 100 would be sufficient because both studies A and B were limited to patients with a longer time of ICU stay [2]. Values are given as medians (25th, 75th percentiles). Pearson’s linear regression was used for correlations, and Mann-Whitney and Wilcoxon tests were used for comparisons between and within groups. Proportions were analyzed by two-sided Fisher’s exact test. Mortality predictors were assessed using univariate logistic regression and stepwise forward multiple logistic regression (NCSS 2007 (Hintze J, NCSS LLC, Kaysville, UT, USA)).

## Results

**Figure 2:**
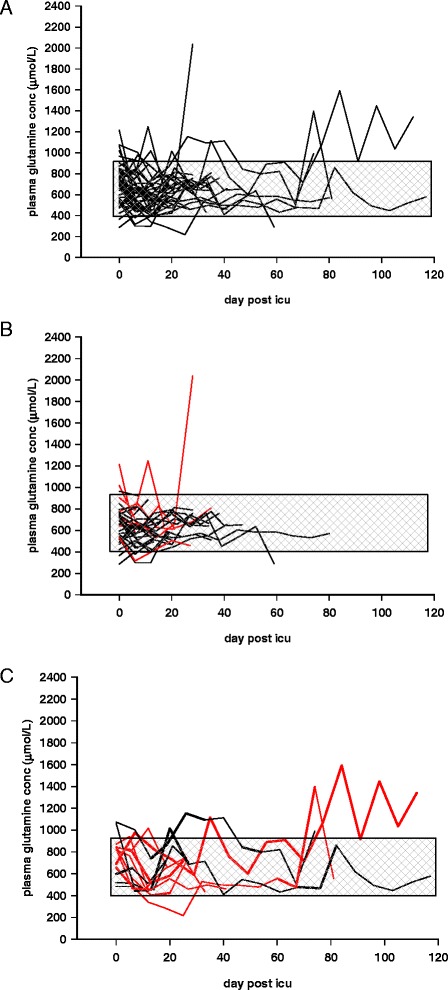
**Post-ICU plasma glutamine concentrations of patients (n = 49), admitted to the ICU and given exogenous intravenous glutamine supplementation together with full nutrition for >3 days.** Patients were sampled on their last day of ICU stay (during ongoing intravenous supplementation) and thereafter every 5 to 7 days during the remaining hospital stay. All patients are depicted in **(A)**. The reference interval used in the study, 400 to 930 μmol/L is indicated by gray shading. **(B)** only illustrates patients who were not readmitted to ICU (n = 35), where the nonsurvivors at 12 months (n = 7) are shown as red lines. **(C)** illustrates patients who were readmitted to the ICU (n = 14), nonsurvivors at 12 months (n = 8) are shown as red lines. Thick lines illustrate periods during which patients in this subgroup were again given intravenous glutamine supplementation during full feeding. ICU, intensive care unit.

In study A (the post-ICU temporal pattern of plasma glutamine concentrations) patients were included between October 19, 2011 and June 11, 2012 (Figure 1A). Sixty-three patients were included to reach the target, which resulted in 49 patients who could be evaluated for their post-ICU plasma glutamine concentrations. For patient characteristics see Table 1, and for ICU discharge, total amino acids and glutamine concentrations see Table 2. Figure 2 shows the plasma glutamine concentration in each patient. In subpanels B and C patients are divided into (i) those who were readmitted or not readmitted to ICU, and (ii) survivors and nonsurvivors. The glutamine supplementation periods are indicated for readmitted patients. In total, 210 post-ICU samples were collected: 6 were <400 μmol/L and 16 were >930 μmol/L. Values outside these limits have been shown to be associated with an increased mortality risk at ICU admission [2]. At discharge from the ICU, plasma glutamine concentration dropped in 30 out of 49 patients from 690 (532,818) to 622 (506,765) μmol/L (*P* = 0.054).

**Table 1:** **Patient characteristics**

	**Gender (male)**	**Age (years)**	**SAPS III**	**LOS (days)**	**SOFA discharge**	**Dialysis**
**Study A (n = 63)**	39 (62%)	64 (56,71)	68 (62,80)	9 (7,14)	4 (2,8)	13 (21%)
**Study A (n = 49)**	31 (63%)	63 (55,72)	68 (60,80)	9 (6,13)	4 (2,7)	10 (20%)
**Survivors (n = 40)**	26 (65%)	63 (56,71)	65 (62,74)	9 (6,13)	3 (2,5)	3 (8%)
**Nonsurvivors (n = 23)**	13 (56%)	65 (61,71)	71 (64,89)	8 (7,14)	7 (4,9)	10 (43%)
***P*** **value**	0.59	0.62	0.042	0.81	0.00085	0.0012
**Study B (n = 100)**	58 (58%)	65 (56,72)	68 (61,77)	9 (6,14)	4 (2,6)	16 (16%)
**Study B (n = 92)**	55 (60%)	65 (56,72)	68 (62,78)	9 (6,13)	4 (2,7)	16 (17%)
**Survivors (n = 54)**	32 (60%)	65 (56,72)	65 (58,70)	8 (6,11)	3 (2,5)	6 (11%)
**Nonsurvivors (n = 38)**	23 (60%)	65 (60,72)	74 (66,88)	10 (6,16)	6 (2,8)	10 (26%)
***P*** **value**	1.00	0.74	0.0013	0.15	0.043	0.09

All subjects included in Study A (n = 63) and subjects with post-ICU samples (n = 49), and all subjects included in Study B (n = 100) and subjects with post-ICU samples (n = 92). Comparison of survivors and nonsurvivors was done in each group using Mann-Whitney or Fischer’s exact tests. SAPS III, simplified acute physiology score III; LOS, length of stay; SOFA, sequential organ failure assessment.

**Table 2:** **Plasma amino acids**

	**Total amino acids (mmol)**	**Glutamine (μmol)**	**% Glutamine**
**Study A (n = 63)**	3.02 (2.61,3.36)	662 (524,796)	22.6 (19.2,24.5)
**Study A (n = 49)**	3.11 (2.63,3.34)	690 (532,818)	22.9 (19.2,26.0)
**Survivors (n = 40)**	2.85 (2.60,3.28)	596 (491,744)	22.5 (19.0,23.5)
**Nonsurvivors (n = 23)**	3.12 (2.75,3.67)	777 (648,848)	23.9 (21.9,26.7)
***P*** **value**	0.12	0.0038	0.08
**Study B (n = 92)**	2.36 (2.02,2.95)	506 (414,658)	21.7 (17.8,24.1)
**Survivors (n = 54)**	2.29 (2.03,2.74)	490 (397,604)	21.3 (17.0,24.4)
**Nonsurvivors (n = 38)**	2.46 (1.98,3.02)	517 (418,675)	22.4 (19.3,23.9)
**Odds ratio (95% CI)**	0.99 (0.65-1.52)	1.001 (0.999-1.003)	1.03 (0.95-1.12)
***P*** **value**	0.86	0.45	0.48

All subjects included in Study A (n = 63) with discharge samples and subjects with consequent post-ICU samples (n = 49), and subjects included in Study B with post-ICU samples (n = 92). Comparison of survivors and nonsurvivors was done in each group using the Mann-Whitney test. CI, confidence interval.

**Figure 3:**
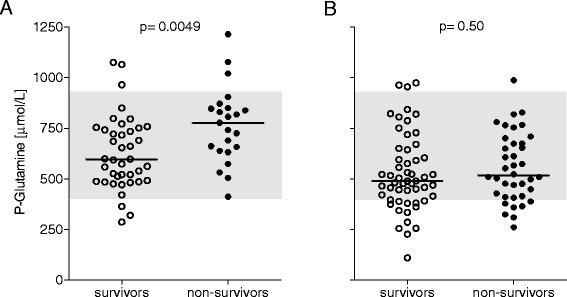
**Plasma glutamine concentrations in ICU patients given exogenous intravenous glutamine supplementation together with full nutrition for >3 days and who were discharged alive from ICU, in relation to 12-month, post-ICU mortality. (A)** illustrates discharge glutamine level (n = 63), and **(B)** illustrates glutamine levels 24 to 72 hours post discharge (n = 92). Discharge glutamine level during ongoing intravenous supplementation but not post-ICU glutamine level was associated with mortality outcome. Gray shading indicates the reference interval used, 400 to 930 μmol/L. Horizontal lines indicate median values. ICU, intensive care unit.

Regarding the samples collected during ongoing iv glutamine supplementation on the last day of ICU stay all 63 patients were evaluated (Tables 1 and 3a,b), there was a difference between 12-month survivors and nonsurvivors, 596 (491,744) versus 777 (648,848) μmol/L (*P* = 0.004) respectively (Figure 3A). There was also a difference in the sequential organ failure assessment (SOFA score on the last day of ICU stay 3 (2,5) versus 7 (4,9) (*P* = 0.0003). Using univariate logistic regression analysis, discharge SOFA score, admission simplified acute physiology score III (SAPS III) score, dialysis, and discharge plasma glutamine concentration were identified as mortality predictors (Table 3a). The stepwise regression analysis indicated that at discharge, the discharge SOFA score complemented the discharge plasma glutamine concentration and enabled the best mortality prediction (Table 3b).

**Table 3:** **Prediction of 12-month mortality in ICU patients that were discharged from ICU after >3 days of iv glutamine supplementation**

a. Univariate logistic regression analysis in group A, patients sampled at discharge and longitudinally post ICU (n = 63)				
	OR (95% CI)		*P*	
Discharge SOFA (per point)	1.38 (1.14-1.67)		0.0003	
Dialysis at discharge	9.49 (2.25-39.9)		0.0008	
Discharge P-glutamine (per μmol/L)	1.004 (1.001-1.007)		0.0046	
Admission SAPS III (per point)	1.041 (1.002-1.081)		0.033	
Discharge total amino acids (per μmol/L)	1.0008 (1.0000-1.0016)		0.042	
b. Stepwise multiple logistic regression analysis in group A, patients sampled at discharge and longitudinally post ICU (n = 63)				
	β	OR (95% CI)	*P*	% Correct
Intercept	−5.45	0.004 (0.0002-0.081)		
Discharge SOFA (per point)	0.335	1.40 (1.13-1.72)	0.0004	73.0
Discharge P-glutamine (per μmol/L)	0.041	1.004 (1.001-1.008)	0.0043	76.2
c. Univariate logistic regression analysis in group B, patients sampled 24–72 hours post ICU (n = 92)				
	OR (95% CI)		*P*	
Discharge SOFA (per point)	1.17 (1.02-1.34)		0.025	
Post ICU P-glutamine (per μmol/L)	1.0008 (0.9985-1.0031)		0.49	
Admission SAPS III (per point)	1.06 (1.02-1.09)		0.0007	

ICU, intensive care unit; iv, intravenous; OR, odds ratio; CI, confidence interval; SOFA, sequential organ failure assessment; P, plasma; SAPS III, simplified acute physiology score III; β, regression coefficient; % correct, fraction correctly predicted by model.

In study B (the post-ICU concentration of plasma glutamine as outcome predictor), patients were included between October 10, 2011 and March 27, 2013 (Figure 1B). The target was 100 patients, out of which 92 could be evaluated, for patient characteristics see Table 1, and for post-ICU total amino acids and glutamine concentrations see Table 2. No difference in post-ICU plasma glutamine concentration existed regarding 12-month, all-cause mortality: 490 (397,604) versus 517 (418,675) μmol/L (*P* = 0.45), for survivors (n = 54) and nonsurvivors (n = 38) respectively (Figure 3B). Post-ICU plasma glutamine concentration was not a significant mortality predictor (Table 3c). In addition, there was no significant correlation between post-ICU plasma glutamine concentration and discharge SOFA score, r = 0.07, (*P* = 0.51). Using univariate regression analysis, discharge SOFA score as well as admission SAPS III were identified as mortality predictors, but not post-ICU plasma glutamine concentration (Table 1c). The stepwise regression analysis indicated that neither discharge SOFA score nor post-ICU plasma glutamine concentration added any prediction value to admission SAPS III score, which gave the best mortality prediction in this group of patients.

## Discussion

The main findings from studies A and B were that post-ICU plasma glutamine levels were within the normal range and that they did not predict mortality. Not unexpectedly, admission SAPS III and discharge SOFA were the strongest mortality predictors in this highly selected subgroup of ICU patients. Remarkably, discharge plasma glutamine concentration during ongoing intravenous supplementation added prediction value - with a higher concentration within normal limits being more unfavorable.

The nutrition protocol was adapted from the protocol used in the Scandinavian Glutamine Trial [12] because hypotheses behind the present protocols were generated from that trial. This, of course, limits the generalizability of the findings. Eligible patients were supplemented intravenously with a glutamine-containing dipeptide. The objective was to normalize plasma glutamine levels. Study A somewhat demonstrated that this objective was achieved. Out of 63 patients sampled on the last day of ICU stay during ongoing glutamine-containing infusing, 56 out of 63 were within the range 400 to 930 μmol/L, 3 were <400 μmol/L and 4 were >930 μmol/L. These limits were chosen from an earlier study when glutamine levels at admission were tested for outcomes prediction [2]. Sometimes the normal range in healthy subjects is given as 500 to 850 μmol/L [13], but we thought the reference to limits that were proven to represent risk at admission (among the studied mixed cases) may be of greater relevance.

The hypothesis of post-ICU plasma glutamine concentration as a mortality predictor could be dismissed in the patient group that required a long ICU stay. Admission SAPS III score was the best mortality predictor in this selected group of patients, also at discharge. From these data, there is no rational to treat patients with glutamine supplementation post ICU. In general, post-ICU plasma glutamine levels were not low, and they did not facilitate mortality prediction. Yet patients with a glutamine deficiency might exist, and determination of plasma glutamine concentrations (or even better, determination of endogenous glutamine production) should identify such patients. Studies in this area might resolve this issue.

Supraphysiological plasma glutamine concentrations are perhaps more common in critically ill patients than earlier thought. In our observational study of admission glutamine levels, concentrations >930 μmol/L were associated with an unfavorable outcome [2]. In acute fulminant liver failure similar supraphysiological glutamine levels are quite common [14]. In an earlier publication, we reported a case with an extremely high concentration, which was normalized upon recovery [15].

In studies A and B, 16 out of 210 post-ICU samples were supraphysiological. Out of these, 11 were during iv glutamine supplementation in the ICU after readmission, and 5 were post ICU without any supplementation. Of the supraphysiological values 7 out of 16 values were recorded in 3 patients who survived and, 9 out of 16 values were recorded in 4 patients who did not survive (Figure 2C). Most importantly, studies are needed to explain the relation between plasma glutamine concentration and the endogenous glutamine production in various states of critical illness. Others have reported a higher plasma glutamine concentration (within normal limits) in nonsurviving long-staying ICU patients, but supraphysiological concentrations of other amino acids were reported to be stronger mortality predictors in that report [16].

Studies A and B were not designed to find out if supraphysiological levels of glutamine may be toxic. The REDOX study reported a more unfavorable outcome in ICU patients - given a hypocaloric diet containing pharmacological doses of glutamine administered enterally and intravenously [9]. But the plasma concentration data from a small subgroup of these patients did not reveal high glutamine values during treatment. So the harm associated with glutamine supplementation in the REDOX study was not associated with high plasma glutamine concentrations in the reported data. In the material from studies A and B, there is a signal from individual subjects that high glutamine levels - regardless of supplementation - may be associated with an unfavorable outcome. More clarity is needed whether plasma glutamine concentration is an indicator of glutamine depletion. Consequently, further studies over glutamine kinetics (production and utilization) in relation to plasma glutamine concentration are needed [17].

Particular interest has been given to kidney failure as a possible association to high glutamine concentrations and an unfavorable outcome [7]. Here we found dialysis to be a mortality predictor at ICU discharge in Study A. However, this statistical relationship was not maintained in the multivariate analysis outside the prediction contained in the discharge SOFA score. It must be emphasized that these *post hoc* analyses are of a purely hypothesis-generating character, and the statistical power contained in these calculations is limited [18,19]. In particular, it may be noted in Table 1 the difference in dialysis at discharge as related to mortality between groups A and B, although there were identical inclusion criteria and a considerable overlap in recruitment between the two groups, underlining the hazards associated with *post hoc* analyses.

The strengths of studies A and B are that consecutive patients were included and that 100% follow-up was possible. The limitations are that the ICU patient case mix may limit the generalizability of the result. Ongoing intravenous glutamine supplementation during ICU stays is also uncommon, but was part of the hypothesis following the result of the Scandinavian Glutamine Trial [12].

## Conclusions

ICU survivors were not low in plasma glutamine after ICU discharge, although no exogenous supplementation was given. In addition, post-ICU glutamine level was not a mortality predictor in the way that admission glutamine has been demonstrated to be. So after ICU discharge, exogenous glutamine supplementation does not have the same rationale as during ICU stay. At ICU discharge a higher plasma glutamine level was associated with post-ICU mortality. The possible indication for exogenous glutamine supplementation during ICU stay is still not properly defined.

## Key messages

- Post-ICU plasma glutamine concentration in ICU survivors is within normal range and it is not an outcome predictor.
- Supranormal plasma concentrations of glutamine were seen more often than expected. The pathogenetic relevance of this finding must be elucidated.

## Abbreviations

CI: confidence interval
HPLC: high-pressure liquid chromatography
ICU: intensive care unit
iv: intravenous
OPA/3-MPA: ortho-phtaldialdehyde/3-mercaptopropionic acid
OR: odds ratio
SAPS III: simplified acute physiology score III
SD: standard deviation
SOFA: sequential organ failure assessment
